# Broad-Band Visually Evoked Potentials: Re(con)volution in Brain-Computer Interfacing

**DOI:** 10.1371/journal.pone.0133797

**Published:** 2015-07-24

**Authors:** Jordy Thielen, Philip van den Broek, Jason Farquhar, Peter Desain

**Affiliations:** Radboud University Nijmegen, Donders Center for Cognition, Nijmegen, Netherlands; Universiteit Gent, BELGIUM

## Abstract

Brain-Computer Interfaces (BCIs) allow users to control devices and communicate by using brain activity only. BCIs based on broad-band visual stimulation can outperform BCIs using other stimulation paradigms. Visual stimulation with pseudo-random bit-sequences evokes specific Broad-Band Visually Evoked Potentials (BBVEPs) that can be reliably used in BCI for high-speed communication in speller applications. In this study, we report a novel paradigm for a BBVEP-based BCI that utilizes a generative framework to predict responses to broad-band stimulation sequences. In this study we designed a BBVEP-based BCI using modulated Gold codes to mark cells in a visual speller BCI. We defined a linear generative model that decomposes full responses into overlapping single-flash responses. These single-flash responses are used to predict responses to novel stimulation sequences, which in turn serve as templates for classification. The linear generative model explains on average 50% and up to 66% of the variance of responses to both seen and unseen sequences. In an online experiment, 12 participants tested a 6 × 6 matrix speller BCI. On average, an online accuracy of 86% was reached with trial lengths of 3.21 seconds. This corresponds to an Information Transfer Rate of 48 bits per minute (approximately 9 symbols per minute). This study indicates the potential to model and predict responses to broad-band stimulation. These predicted responses are proven to be well-suited as templates for a BBVEP-based BCI, thereby enabling communication and control by brain activity only.

## Introduction

A Brain-Computer Interface (BCI) enables one to control a device by using brain activity only, bypassing the peripheral nervous system. Because BCI is not dependent on muscle control, it provides an additional output channel for the brain to be used for communication and control.

**Fig 1:**
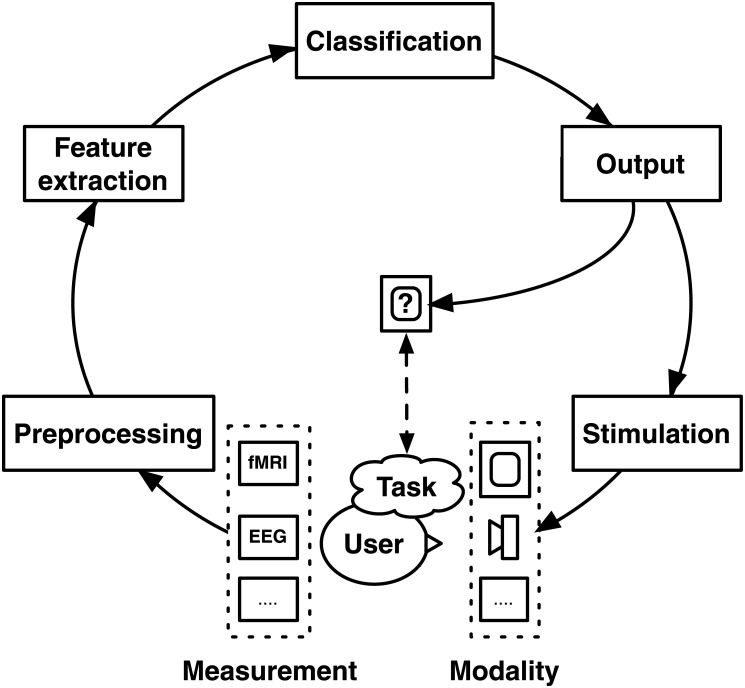
The BCI cycle. A general framework of BCIs, showing the consecutive steps in signal processing. These steps involve stimulation in a specific modality, data measurement and pre-processing, data analysis, and output or feedback generated by the BCI. Adapted from [1].

The online BCI cycle can be initiated by specific stimulation that evokes characteristic task-related brain activity. During stimulation, brain activity is commonly measured by electroencephalogram (EEG) recordings. A computer then interprets the measured EEG following several pre-processing steps (e.g., cleaning the data) and machine learning techniques (e.g., learning task-related and subject-specific brain activity). With this analysis a computer can decode and detect task-related information from brain activity. The output is usually fed back to the user after which a new cycle starts. The cyclic diagram as shown in Fig 1 forms a general framework against which most BCI research can be positioned [1].

In this study we focus on a visual matrix speller, first proposed by [2]. A typical visual matrix speller presents a grid to the user, each cell containing one symbol. During stimulation, these cells alter in luminance (i.e., they flash). By visually attending a cell, a cell-specific Visually Evoked Potential (VEP) is elicited, which is detectable by a BCI and enables the selection of individual symbols. At least five different stimulation paradigms can be used to produce such recognizable brain patterns (for a review see [3]). Commonly, an oddball sequence is used that evokes a distinct time-locked Event-Related Potential for targets (i.e., Time Domain Multiple Access, e.g., see P300-based speller [4]), or periodic flicker that evokes distinct Steady-State Visually Evoked Potentials (i.e., Frequency Domain Multiple Access, e.g., see SSVEP-based speller [5]). Instead, in this study we focus on Code Division Multiple Access schemes for stimulation. These schemes minimize the cross-stimuli interference by making the cell-specific stimulation patterns (i.e., the sequences of flashes) orthogonal to each other. This is particularly achieved by using pseudo-random noise-codes. In the visual domain these can be applied as flash sequences that evoke specific Broad-Band Visually Evoked Potentials (BBVEPs). The BBVEP-based speller, though uncommon in current literature, has outperformed spellers that use more common stimulation schemes [6].

The BBVEP-based matrix speller was first proposed by [7] and tested with an ALS patient using intracranial electrodes and an 8 × 8 grid. The patient could write up to 10–12 words per minute [8]. More recently, the BBVEP-based matrix speller has been adapted to increasingly successful use with EEG recordings. [6] used one 63-bit m-sequence for stimulation. Essentially, multiple responses to this bit-sequence were acquired and averaged to obtain a template. Both bit-sequence and template are circularly shifted in time to create stimuli and templates for multiple classes. New data is classified by selecting the best matching template by means of highest correlation. With a 4 × 4 grid and pre-selected EEG channels, [6] reached a communication rate of 92.8 bits per minute. By deriving optimal spatial filters with Canonical Correlation Analysis (CCA) and a 4 × 8 grid, this communication rate was improved to 108 bits per min [9]. Instead of a simple averaging approach to create templates, a Once Class Support Vector Machine (OCSVM) can better estimate the probability distribution of high-dimensional data. Applying a OCSVM together with CCA resulted in a communication rate of 133.6 bits per minute [10]. This communication rate was improved to 144 bits per minute—the highest speed reported so far—by implementing an adaptive classifier [11].

In this study, we investigate a novel approach to synchronous BBVEP-based BCI. First, contrary to an m-sequence, Gold codes are used. Gold codes are pseudo-random bit-sequences generated by combining two carefully selected m-sequences [12]. A set of Gold codes contains numerous bit-sequences that have an optimized (i.e., minimized) cross-correlation. Thus, Gold codes can be directly applied to classification problems with a higher number of classes. Additionally, a Gold code has an optimized auto-correlation enabling their use in an asynchronous BBVEP-based BCI (i.e., continuous stimulation without any phase-lock). A set of m-sequences has no guaranteed good cross-correlation. However, any m-sequence does have a good auto-correlation which makes it possible to create a set of circular-shifts of the same m-sequence (e.g., [6, 10, 11]). A set of circular-shifted m-sequences thus exhibits good cross-correlation, but the possibility for asynchronous use is lost.

Second, in this study the EEG responses to these Gold codes are modeled with a generative model. This generative model, referred to as reconvolution, can be trained quickly in order to generate template EEG responses to bit-sequences [13]. To put reconvolution to the test, two sets of Gold codes are used. The first set is used for stimulation during the training phase of the BCI. Recorded responses to this first set are used to train reconvolution, in order to predict responses to the second set. This second set is used for stimulation during the testing phase of the BCI. Thus, a clear separation exists between training and testing, which will only succeed if reconvolution can truly generate and predict responses well.

Third, with reconvolution EEG responses to any bit-sequence can be predicted, either seen (i.e., used during training) or unseen. Though, potentially only a subset of stimulation sequences is required. Therefore an optimization pipeline is possible: a subset of codes is selected, allocated to the speller grid according to the dissimilarity of their predicted responses. Additionally, an early stopping algorithm is defined [14]. To summarize, the contribution of this study is three fold: (1) validation of a BBVEP-based matrix speller using Gold codes, (2) validation of a generative model for template generation and prediction and (3) optimization of the speller design.

## Methods

### Participants

Twelve university students (4 male, mean age 24.0 years, standard deviation 2.3 years) participated in the experiment after pre-screening and providing written informed consent. Pre-screening consisted of excluding participants with any history of epilepsy. All participants had normal or corrected-to-normal vision and reported no central nervous system abnormalities. After the experiment participants were paid for their contribution to this study. The experiment was approved by the Ethical Committee of the Faculty of Social Sciences at the Radboud University Nijmegen.

### Equipment

The speller was presented on a 24 inch BenQ XL2420T LED monitor with 120 Hertz refresh rate and a resolution of 1920 × 1080 pixels. Timing accuracy was measured with a light-sensor and was always within 2 ms. The light intensity of white, black and grey were 185 lux, 4 lux and 55 lux, respectively.

Participants were seated in a chair at a distance of 75 cm from the monitor. The height of the chair was adjusted so that the participant was facing the center of the monitor.

EEG data was recorded with 64 sintered Ag/AgCl active electrodes according to the 10–20 system, amplified by a Biosemi ActiveTwo amplifier. Data was acquired at 2048 Hertz, though the data was immediately down-sampled to 360 Hertz to match a multiple of the stimulation frequency (i.e., the 120 Hertz monitor refresh rate). Data was pre-processed following linear de-trending, common average referencing, and spectral filtering with two pass-bands at 5 − 48 Hertz and 52 − 100 Hertz. Processed data is available at DANS (http://dx.doi.org/10.17026/dans-zth-37cr, [15]). The raw EEG data is stored at the Donders Center for Cognition, and can be requested by e-mail (info@donders.ru.nl).

### Stimuli

**Fig 2:**
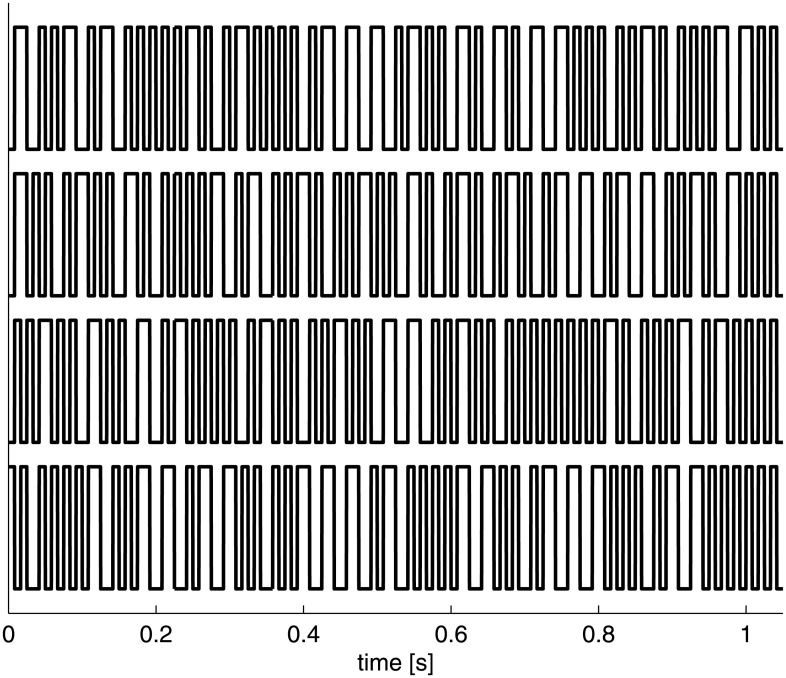
Stimulation sequences. Four modulated Gold codes are shown of *l* = 2*(2^*m*^ − 1) = 126 bits. These are pseudo-random binary-codes that are sequences of short on-off runs (i.e., ‘10’ or ‘100’) and long on-off runs (i.e., ‘110’ or ‘1100’). These on-off runs are used to modulate the luminance of the cells, and thus represent short and long flashes.

The cells in the 6 × 6 speller grid were presented either black or white. The flash sequences could thus be represented as binary sequences where ones and zeros represented white and black cells respectively. In this study two sets of Gold codes were used [12]. The first set, denoted *V*, was used for training and is generated with a linear feedback shift register of length *m* = 6 and feedback taps at positions 6,5,2,1 and at 6,1 (for the generation process see [12, 16]). The second set, denoted *U*, was used for testing and was generated with the same register, but with feedback taps at positions 6,5,3,2 and at 6,5. Both *V* and *U* were modulated by xor-ing them with a double-frequency bit-clock, which limited low-frequency content. *V* and *U* contained *n* = 2^*m*^ + 1 = 65 bit-sequences that all had a length of *l* = 2*(2^*m*^ − 1) = 126 bits. One such bit-sequence had a duration of *t*_*b*_ = 126/120 = 1.05 s. These modulated Gold codes were sequences of short on-off runs (i.e., ‘10’ or ‘100’) and long on-off runs (i.e., ‘110’ or ‘1100’), representing short and long flashes, respectively. These two flashes were of length *t*_*s*_ = 1/120*1000 = 8.3 ms and *t*_*l*_ = 2/120*1000 = 16.6 ms, respectively. For an illustration of these modulated Gold codes, see Fig 2.

### Calibration

A template matching classifier was constructed to identify the attended cell. In template matching, the best fitting template is chosen using a similarity measure. In this study we used correlation to measure the similarity between a single-trial and several templates, which is maximized to obtain a class-label:

$$\begin{array}{c} y=\arg\max_{i}\frac{x^{⊤}T_{i}}{\sqrt{x^{⊤}x\cdot T^{⊤}T}} \end{array}$$ (1)

where *y* is the predicted class label, *x* is the single-trial and *T*_*i*_ is the template of class *i*.

**Fig 3:**
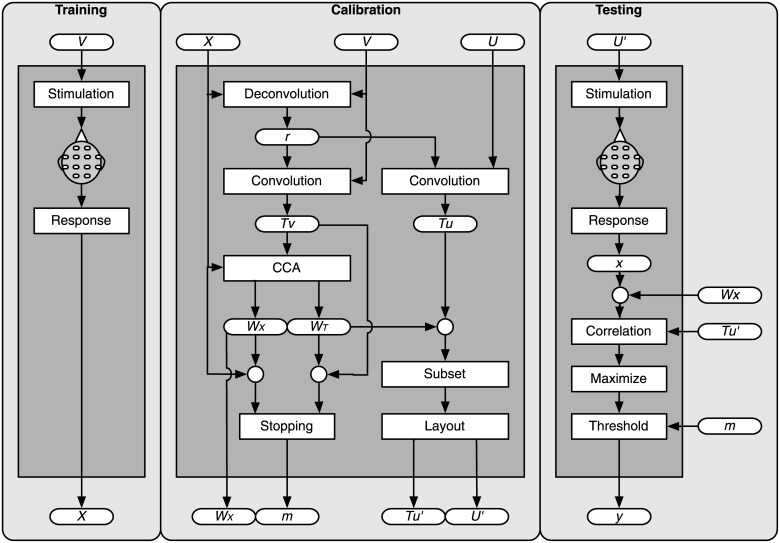
The online pipeline. Three stages exist: training, calibration and testing. During training, responses *X* to stimuli from *V* are recorded. During calibration, *X* is deconvolved to pulse responses *r* using *V*. Template responses *T*_*V*_ and *T*_*U*_ are generated by convolving these *r* with the bit-sequences *V* and *U*, respectively. Templates are multiplied (circles) with filters (*W*_*X*_, *W*_*T*_) designed by CCA. The subset and layout are optimized giving *U*′ and $T_{U}^{\prime }$, and stopping margins *m* are learned. In the testing phase, a new single-trial *x* is assigned the class-label *y* that maximizes the correlation between the spatially filtered single-trial *x* and templates $T_{U}^{\prime }$. The classifier emits the class-label if the maximum correlation exceeds the threshold margin. In the case wherein the margin is not reached, more data is collected.

The classifier, including the templates, was constructed following five consecutive steps: template generation, spatial filtering, subset optimization, layout optimization, and learning of stopping criteria (see Fig 3). Matlab routines are available at GitHub (https://github.com/thijor/Reconvolution).

#### Template generation: Reconvolution

To be able to identify the attended cell using template matching, templates *T*_*i*_ are required. In order to obtain these, *k* trials were recorded while bit-sequences from *V* were presented. Normally, averaging trials of one class would yield a template. However, this would require many trials to cover all bit-sequences (i.e., say 100 trials for each of 65 bit-sequences), which is infeasible. Instead, we defined a generative method for template generation: reconvolution.

**Fig 4:**
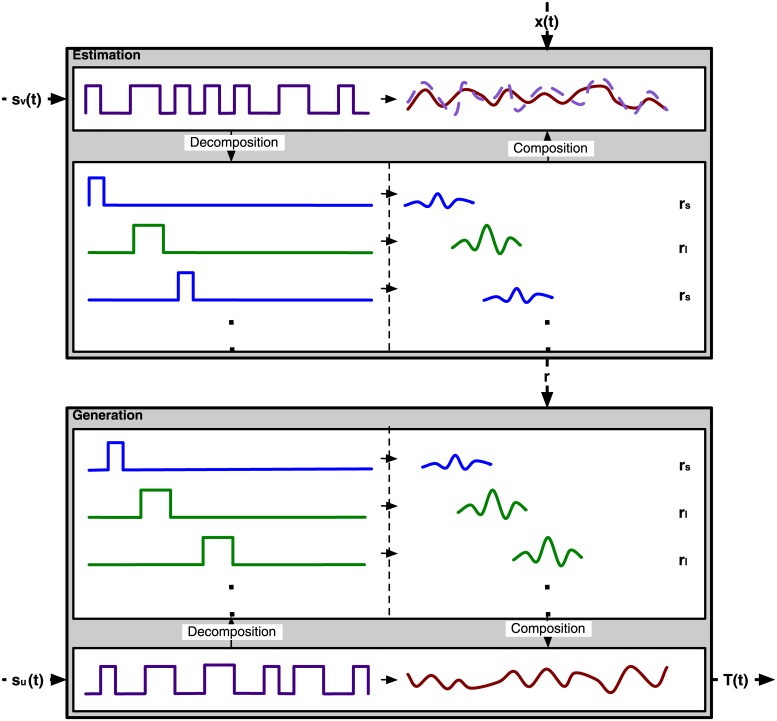
Reconvolution. Reconvolution is a two-step approach: estimation (top) and generation (bottom). In the first step a recorded response *x*(*t*) is decomposed according to the structure in a bit-sequence *s*_*v*_(*t*). The decomposition yields transient responses *r*_*s*_ and *r*_*l*_ to short and long pulses, respectively. In the second step, these estimated responses can be used to generate or predict the response *T*(*t*) to a (unseen) bit-sequence *s*_*u*_(*t*).

We exploit the fact that the modulated Gold codes are built as a sequence of only two events: short and long flashes. Assuming linearity of the brain response, the response to a sequence of flashes can be found by convolution: adding time shifted versions of single-flash responses. Conversely, these single-flash responses can be found by deconvolution, which boils down to linear regression. Brain processes behave non-linearly as well, though assuming linearity has proven to be sufficient for modeling of specific early visual responses (e.g., see [17]). Reconvolution is a two step approach for template generation combining deconvolution and convolution (see Fig 4). First, responses to individual flashes are estimated by decomposing full-responses (i.e., the estimation-step). Second, full-responses to (un)seen bit-sequences are generated by applying the estimated single-flash responses (i.e., the generation-step).

The first step in reconvolution is estimation. Acquired data is decomposed into a linear combination of a structure matrix and responses. In [18], a study on auditory noise-tagging, responses to rising (‘01’) and falling (‘10’) events in the bit-sequence were estimated. In this study, the decomposition is based on short (i.e., ‘10’ or ‘100’) and long flashes (i.e., ‘110’ or ‘1100’). We call the responses to these single-flashes pulse responses, not to be confused with impulse responses (to Dirac impulses). The generic model of decomposition for a single-channel single-trial can be written as:

$$\begin{array}{c} \tilde{x}\left(t\right)=\sum_{\tau =1}^{L}I_{s}\left(t\right)r_{s}\left(t-\tau \right)+I_{l}\left(t\right)r_{l}\left(t-\tau \right) \end{array}$$ (2)

where $\tilde{x}\left(t\right)$ is a modeled full-response at time *t*, *I*_*s*_(*t*) and *I*_*l*_(*t*) are indicator functions that have the value 1 if there is a short/long flash at time *t* and 0 elsewhere, *r*_*s*_(*t*) and *r*_*l*_(*t*) are the pulse responses to short/long flashes at time *t*, and *L* is the length of both *r*_*s*_ and *r*_*l*_. This relationship can be expressed in a more compact matrix notation:

$$\begin{array}{c} \tilde{x}=M_{s}r_{s}+M_{l}r_{l}=[\begin{array}{cc} M_{s} & M_{l} \end{array}][\begin{array}{c} r_{s}\\ r_{l} \end{array}]=Mr \end{array}$$ (3)

**Fig 5:**
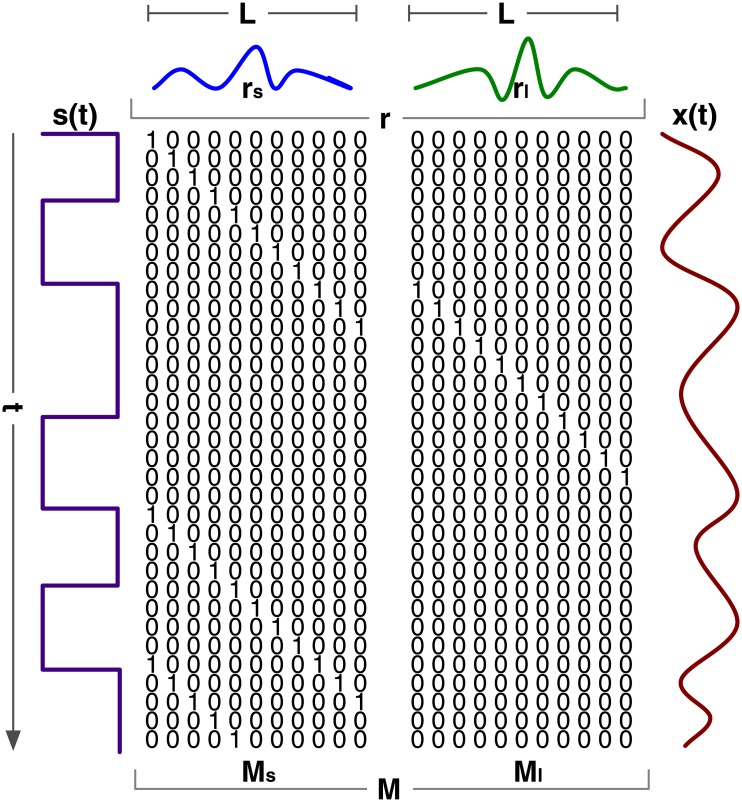
Structure Matrix. In reconvolution a structure matrix **M** is used that indicates when a certain pulse happens in a bit-sequence *s*(*t*). **M** is the concatenation of *M*_*s*_ and *M*_*l*_, both having the value 1 whenever a short/long pulse happens at time *t*, shifting down *L* rows. This matrix is used to (de)compose a full-response *x*(*t*) to pulse responses *r*_*s*_ and *r*_*l*_ to short/long flashes.

Here, $\tilde{x}$ is a column vector of *s* samples. *M*_*s*_ and *M*_*l*_ are structure matrices that have the value 1 in column 1 at row *t* if there is a short/long flash at time *t*. For columns 2 to *L* ones shift down to *t* + *L*. *M*_*s*_ and *M*_*l*_ are 0 in all other locations. *M*_*s*_ and *M*_*l*_ are both of size *s* × *L* and thus *M* is of size *s* × (2⋅*L*). An example of a structure matrix is illustrated in Fig 5. Both *r*_*s*_ and *r*_*l*_ are column vectors of *L* samples and thus *r* is of size (2⋅*L*) × 1. This model can be generalized to multiple single-channel single-trials by concatenation, to form the following linear regression problem:

$$\begin{array}{c} X=[\begin{array}{ccc} x_{1} & \cdots & x_{k} \end{array}]=[\begin{array}{ccc} M_{1} & \cdots & M_{k} \end{array}]r=Mr \end{array}$$ (4)

where *X* is the concatenation of *k* single-channel single-trials ((*s*⋅*k*) × 1), and **M** is the concatenation of all corresponding *M* ((*s*⋅*k*) × (2⋅*L*)). This means that *r* can be found solving the linear equation; the solution can be found as follows:

$$\begin{array}{c} r=M^{+}X \end{array}$$ (5)

where **M** ^+^ is the pseudo-inverse of **M**.

The second step in reconvolution is generation. Recall that the *k* single-trials used stimulation sequences from *V*, hence the full-responses to bit-sequences in *V* can be generated, denoted as templates *T*_*V*_. Since *r* is estimated, any bit-sequence that is built up from the same events (here short and long flashes) can be predicted by constructing its structure matrix *M* and multiplying it with the response vector *r*. Thus, all responses to bit-sequences in *U* can be predicted as well, denoted as templates *T*_*U*_:

$$T_{V}=M_{V}r$$ (6)

$$T_{U}=M_{U}r$$ (7)

When the estimation and generation steps are repeated for each channel, reconvolution generates *T*_*V*_ and *T*_*U*_ containing the (predicted) templates for *V* and *U* respectively. Both *T*_*V*_ and *T*_*U*_ have dimensions *c* × *s* × *n* (i.e., channels by samples by templates).

#### Spatial filtering: Canonical Correlation Analysis

EEG is a noisy signal. To increase the signal to noise ratio (SNR), a noise reduction method such as spatial filtering is often applied. A spatial filter projects the signals from sensor space into source space, where sources are separated by any criterium. For each component, the filter combines electrode signals into a weighted sum. This multivariate approach usually outperforms feature selection by channel picking (e.g., [6, 10]). Here we use Canonical Correlation Analysis (CCA). In short, this works by estimating a spatial filter, which spatially (i.e., across EEG channels) averages (i.e., a weighted average) each single-trial time-point to estimate a component’s signal strength. This component’s signal strength is as close as possible to the signal strength estimated for the same time-point when temporally averaging all trials. CCA does this by deriving two spatial filters *W*_*X*_ and *W*_*T*_, in order to maximize the correlation between $W_{X}^{⊤}X$ and $W_{T}^{⊤}T$:

$$\begin{array}{c} W_{X},W_{T}=\arg\max_{W_{X},W_{T}}\frac{W_{X}^{⊤}XT^{⊤}W_{T}}{\sqrt{W_{X}^{⊤}XX^{⊤}W_{X}\cdot W_{T}^{⊤}TT^{⊤}W_{T}}} \end{array}$$ (8)

Here, *X* represents the concatenated single-trials of size *c* × (*s*⋅*k*). *T* represents a matrix of similar dimensionality, though it contains the corresponding generated templates *T*_*V*_. CCA derives multiple uncorrelated components for both *W*_*X*_ and *W*_*T*_, sorted on canonical correlation. Because we want to optimize the correlation between single-trials and corresponding templates, the first component suffices. The templates are spatially filtered with *W*_*T*_ leaving *T*_*V*_ and *T*_*U*_ to be of size *s* × *n*. The data is spatially filtered with *W*_*X*_ leaving *X* of size *s* × *k*.

#### Subset optimization: Platinum subset

**Fig 6:**
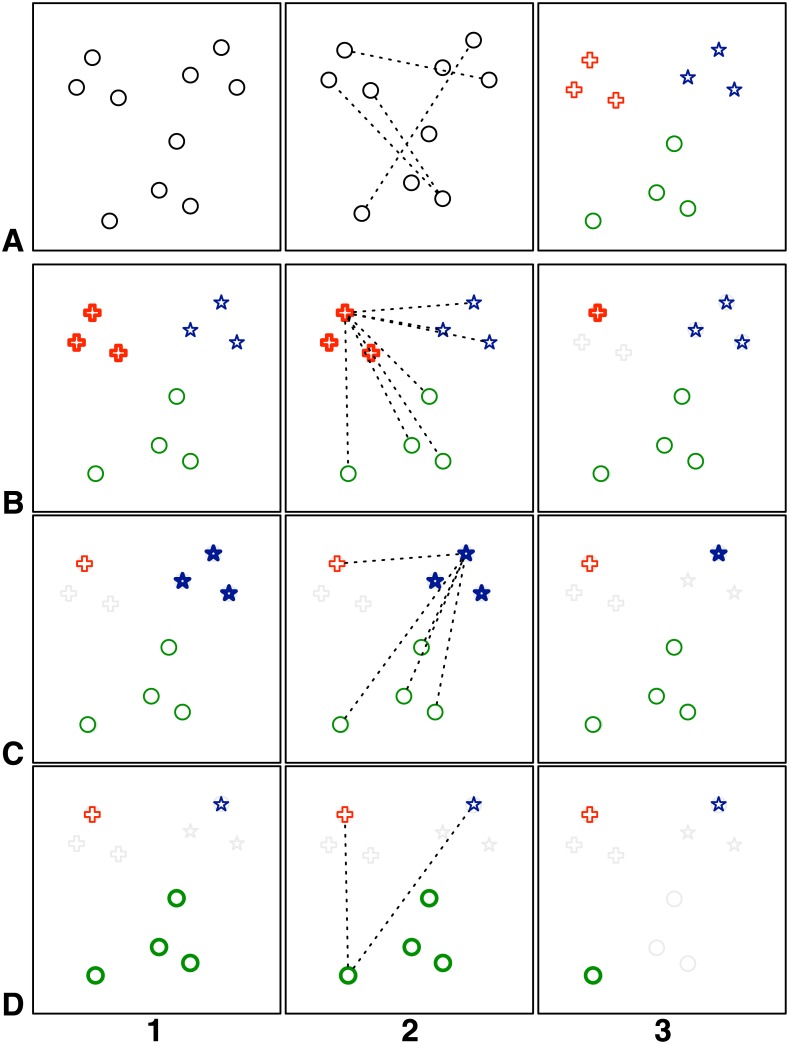
Platinum subsets. The steps for finding a subset of *p* = 3 codes from a set of *n* = 10 codes is shown. First the full set is clustered grouping similar points (A_1_ till A_3_). Then, iteratively (B till D) each cluster is collapsed into a single point by selecting one candidate. This candidate is chosen by maximizing the distance to all other living points outside the cluster (B_2_, C_2_, D_2_). The remaining points form the Platinum subset (D_3_).

*U* contains *n* = 65 codes and for each code, the responses *T*_*U*_ are predicted. In designing a BCI user interface sometimes only *p* codes are needed, where *p* ≤ *n*. Note that while the stimuli are all uncorrelated as binary signals, this does not imply that the responses are uncorrelated too. Therefore a selection process aims at finding an easily response-distinguished subset of codes. Because *T*_*U*_ is known, it is possible to calculate the full cross-correlation matrix of responses. However, choosing a subset of *p* codes from a set of *n* codes by exhaustive search is infeasible, unless *p* is close to 1 or *n*. The combinatorial explosion is harnessed in two steps: clustering and selection (see Fig 6).

First, responses are hierarchically clustered with single linkage to minimize within-group correlation and maximize between-group correlation [19]. Then, *k* clusters are obtained by trimming the clustering-tree to yield *k* leaves.

Second, the best cluster-defining codes are selected in a greedy way. The code from within a cluster that minimizes the maximum response-correlation with all codes outside the cluster, is chosen as the best candidate for a cluster. The cluster is then cropped to this one candidate. This selection process starts with a random cluster and is iterated over all clusters until all contain only one code. These remaining codes form the Platinum subset *U*′ ∈ *U* containing *p* codes. In addition, $T_{U}^{\prime }\in T_{U}$ contains only *p* templates. In this study, *p* = 36 as we use a 6 × 6 matrix for the speller application.

#### Layout optimization

The stimuli are arranged in a 6 × 6 matrix of cells. Depending on the visual angle, codes used for neighbouring cells may leak through in the responses. An optimal layout allocates codes to cells in such a way that neighbouring cells do not correlate much in their responses. For a 6 × 6 speller matrix, given the Platinum subset of 36 codes, an optimal layout cannot be selected by exhaustive enumeration. Instead, a greedy optimization is used.

**Fig 7:**
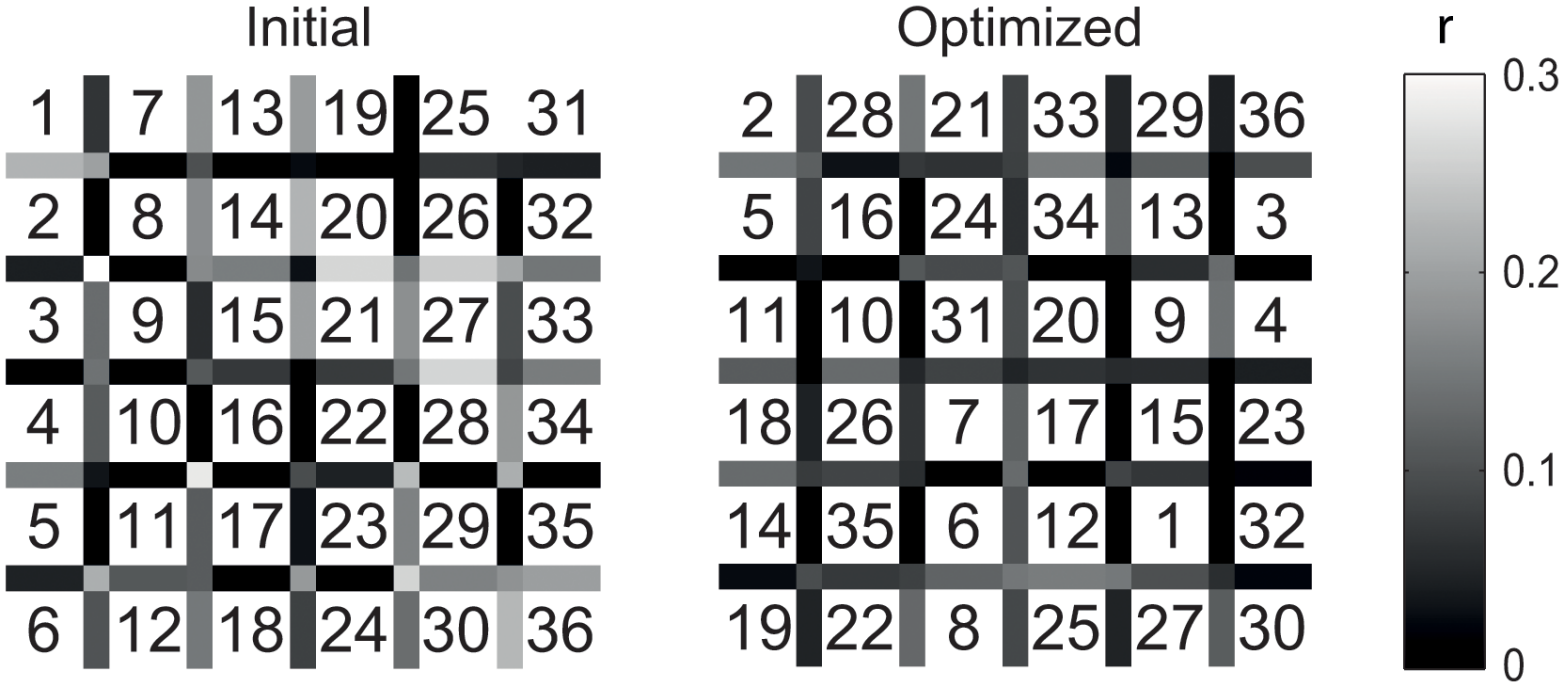
Layout optimization. To prevent cross-talk between neighbouring cells the allocation of bit-sequences to cells is optimized. An initial (left) and optimized (right) layout are shown. Numbers indicate bit-sequences. The shade indicates the correlation between responses to codes from neighbouring cells. The correlations are depicted between horizontal, vertical, and diagonal neighbours. For diagonal neighbours, the maximum correlation of the two diagonals is shown. In this perspective darker colours represent less correlation and better neighbours, hence an increased potential to distinguish.

The algorithm starts with an arbitrary allocation of codes to matrix cells. Then, the worst neighbouring code-pair is selected by means of largest pair-wise correlation in neighbouring templates from *T*_*U*_. The algorithm searches exhaustively for the best exchange of two codes that gives the least pair-wise correlation between any vertical, horizontal, or diagonal neighbours. The algorithm performs the exchange and continues with finding the next worst neighbour until the exchange does not result in a better layout. Because global optimality is not guaranteed, the algorithm is restarted with several random initial layouts. From these optimized layouts, the layout with the lowest maximal pair-wise correlation in neighbouring templates is selected. The improvement of such a layout compared with an initial one is depicted in Fig 7. Both *U*′ and $T_{U}^{\prime }$ are ordered according to this optimized layout.

#### Learning of stopping criteria

When enough training data is available, one can determine a fixed time interval needed for a desired classifier performance. On the contrary, one could also define a criterion to dynamically decide when trials can be stopped, called early-stopping (for an overview see [20]). In online processing of new data, the length of a single-trial is sequentially increased as long as the classifier produces a criterion value below a required margin. If the classifier is sufficiently certain, the trial stops and the classifier presents its output. This approach provides a possible speed gain and an easy adaptation to occasional distractions.

Here, the margin—the correlation difference between best and second best matching templates—is used to indicate that a decision on the best matching template can be made reliably. The algorithm learns these stopping margins *m* based on the evolving distributions of correlation values conditioned on the correctness of outcome. Moreover, the full cross-correlation matrix of the *k* trials from *X* with the templates in *T*_*V*_ is computed. Then, the difference between the best and second best matching template is estimated and separated in two distributions: one containing correct trials (i.e., the best matching template was the correct one), and the other containing incorrect trials. Using these distributions a threshold margin is learned by lowering it until a desired performance is reached. This analysis is repeated for each possible trial length independently.

In our matrix speller the maximum trial length was 4.2 seconds. This trial length started with a segment of 100 milliseconds and increased with 100 milliseconds each iteration. Hence, *m* contains 42 learned stopping margins. These margins were fitted with an exponential function; the first five margins (i.e., the first 500 ms) were artificially set to 1 to enforce a minimal length of data; the last margin (i.e., at 4.2 s) was set to 0 to force a decision at the maximum trial length.

**Fig 8:**
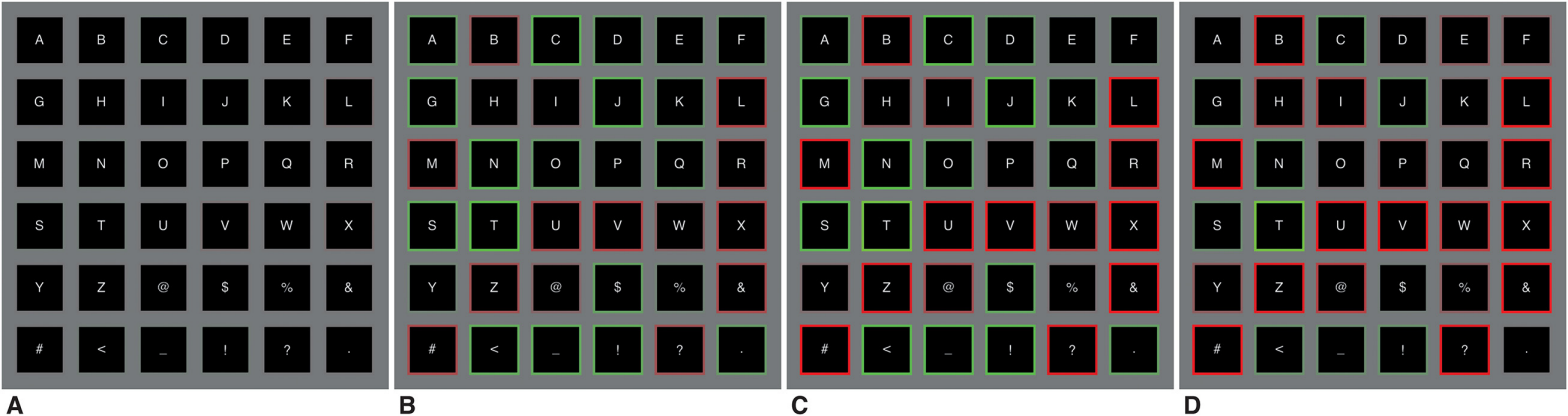
Colour feedback. While a trial progresses, colour feedback is given regarding the classifier’s certainty. All cells start gray (A). A cell is coloured more green if the cell is more likely to be selected, whereas a cell is coloured more red when it is likely to not be selected. The colours are scaled to the specific margin and the maximum and minimum correlation between the single-trial and templates. Here, the target was ‘T’.

These margins *m* were also used to provide the user with feedback regarding the classifier’s certainty. This was communicated by outlining the edges of all cells with a specific colour. At the start of a trial all edges were coloured gray. As the trial progresses, edges were coloured green whenever the cell-specific template reached the margin. Conversely, the cell-specific templates that did not reach the margin were coloured red. The colouring was scaled to the margins and the minimum and maximum correlation to better visualize which cells were most and least likely to be chosen. Because of the scaling, multiple cells could be coloured green, though the highest correlating cell is the greenest. Fig 8 shows four chronological snap-shots of a trial in which the target was ‘T’.

### Experiment

Each participant performed the experiment in one session which was separated in four blocks. In chronological order, the experiment consisted of: a practice block, containing two runs; a training block, containing one run; a copy-spelling block, containing six runs; and a free-spelling block, containing one run. In between the training and copy-spelling block, the template matching classifier was calibrated according to the full pipeline, as described above.

In all runs –except in the free-spelling block– participants were asked to focus at each symbol in the 6 × 6 matrix once, therefore a run contains 36 trials. The order of symbols was randomized, thus participants were performing a random copy-spelling task. A trial started by colouring the target-cell green for 1 second. After target instruction all symbols flashed according to their specific bit-sequence immediately for 4.2 seconds (i.e., four code repetitions). Directly after stimulation, the output of the classifier was presented by colouring the cell blue for 1 second. After this feedback, the next trial was initiated immediately.

In the practice block, the first thirty-six codes from code-set *V* were used. Here, stimulation was adjusted in such a way that the target-cell was always flashing with the same bit-sequence. This was achieved by switching a predefined practice code with the instructed code for each trial. In this way, the participant always focused on the first code from *V* in the first run and the second code from *V* in the second run. The practice block was used to familiarize the participant with the task and the concept of a visual speller. In addition, via these two runs, multiple trials were acquired of two bit-sequences that were used for offline analysis, discussed later on. Because no classifier was yet defined, feedback was always at the instructed cell.

In the training block, also the first thirty-six codes from *V* were used. Thus, the participant attended to each bit-sequence in this subset once. Because no classifier was yet defined, feedback was always as if correct. These *k* = 36 trials were used to calibrate the classifier.

In the copy-spelling block, the bit-sequences from *U*′ were used for stimulation. The copy-spelling block was divided in two conditions: fixed-length and early-stopping. Both conditions contained three runs. In the fixed-length runs, stimulation was always 4.2 seconds long. In the early-stopping runs stimulation was stopped whenever the classifier could make a decision based on the margins *m*. The order of the six runs was randomized over participants, though participants were always told which condition followed next.

The free-spelling block was similar to the copy-spelling block, though was always in early-stopping mode. During this run there were no targets instructed, but still 1 second was reserved for making up the next decision. Participants could maximally spell 50 symbols, after which the speller stopped automatically. By selecting the ‘_’ symbol a space was written; the ‘<’ functioned as a backspace; the ‘#’ had to be selected twice in a row to exit the speller; by selecting either a ‘!’, ‘?’ or ‘.’ twice in a row, text-to-speech software was used to pronounce the sentence, after which the sentence was cleared.

### Validation of BCI performance

Validation of the online performance of the BCI is estimated by the accuracy and speed on all random copy-spelling trials, and all free-spelling trials. The accuracy is defined as the percentage of correctly classified trials. The speed is measured as the Information Transfer Rate (ITR) [21]. In addition to the ITR, the Symbols Per Minute (SPM) measure is used to approximate a more practical speed, as it incorporates the necessity to perform a backspace after an incorrect selection [22]. The measures are as follows:

$$B=\log_{2}N+P\log_{2}P+\left(1-P\right)\log_{2}\frac{1-P}{N-1}\;[bits]$$ (9)

ITR=(T/60)*B[bits/min] (10)

SPM=(T/60)*(P-(1-P))[sym/min] (11)

where *N* is the number of classes, *P* the accuracy in a range of 0 to 1, and *T* the number of seconds required for spelling one symbol. *T* includes both trial time and inter-trial time. In the experiment, the inter-trial time was always 2 seconds (i.e., 1 second instruction and 1 second feedback).

### Validation of reconvolution

The generative power of reconvolution is estimated by means of the explained variance. More specifically, the more variance in the data is explained by reconvolution, the better the template is predicted. Here we chose to compare the predicted templates from reconvolution with ERPs. The practice block data, acquired for this purpose, provides the necessary multiple trials for both modulated Gold codes to be able to construct ERPs.

In both runs in the practice block, *k* = 36 trials were collected. These sets are denoted *X*_1_ for code 1 and *X*_2_ for code 2, both with dimensions *c* × *s* × *k*. Here, *s* contains four code repetitions. Both sets are sliced to single code repetitions, so both *X*_1_ and *X*_2_ are of size $c\times \frac{s}{4}\times (k\cdot 4)$.

The validation of reconvolution is done using *k*-fold cross-validation. Both *X*_1_ and *X*_2_ are split into *k* folds. Reconvolution is applied to both *X*_1_ and *X*_2_ separately, using one fold. This gives two templates matrices *T*_1_ and *T*_2_, both of size $c\times \frac{s}{4}\times 2$. The remaining *k* − 1 folds are used to construct the ERPs by averaging in *X*_1_ and *X*_2_ separately. This gives two ERPs *R*_1_ and *R*_2_ of size $c\times \frac{s}{4}$. Now the explained variance can be estimated by computing the squared correlation between the prediction and the ERP. This is done for seen sequences (i.e., self-prediction, e.g., *T*_1_ predicts *R*_1_) and unseen sequences (i.e., cross-prediction, e.g., *T*_1_ predicts *R*_2_). Validation is performed for each electrode individually and for spatially filtered responses using CCA as defined before.

### Validation of optimizations

Three optimizations were performed: subset optimization, layout optimization, and early-stopping.

To validate the effectiveness of subset optimization, the training block data is used to construct templates *T*_*i*_ for all codes. A paired t-test is then performed between several random subsets and the Platinum subset. Here, dependent variables are the maximum, mean, and minimum correlation within a subset.

To validate the effectiveness of layout optimization, the training block data is used to construct the Platinum subset. A paired t-test is then performed between several random layouts and the optimized layout. Here, dependent variable are the maximum, mean, and minimum correlation within a subset.

To validate the effectiveness of early-stopping, the ITRs as computed in the validation of the BCI performance are considered. A paired t-test is performed to test for any significant effect between the two conditions: fixed-length and early-stopping.

## Results

### Validation of BCI performance

In the copy-spelling block, the average classification rate in the fixed-length condition was 86 percent. This yields an ITR of 38.12 bits per minute and an SPM of 6.93 symbols per minute. In the early-stopping condition, the average classification rate remained 86 percent but on average data segments of 3.21 seconds were sufficient to make a valid decision. This yields an ITR of approximately 48.37 bits per minute and an SPM of 8.99 symbols per minute. Recall that early-stopping was always trained using a targeted classification rate of 95 percent, which was not feasible for all participants. Note that the speed goes up for almost all participants when early-stopping is used. In the calculation of the ITR and SPM an inter-trial interval of 2 seconds was always incorporated. The results on the random copy-spelling task are summarized in Table 1.

**Table 1:** Copy-spelling performance. Performance rates of all individual participants and the grand average during random copy-spelling. Accuracies (P) are given in percentages of correct classifications together with the average trial time needed for classification (including overhead: inter-trial time). The Information Transfer Rate (ITR) and Symbols Per Minute (SPM) are estimated (including the inter-trial time). Note that the early-stopping pipeline was trained with a fixed targeted accuracy of 95 percent for all participants.

	Fixed-Length				Early-Stopping			
	P	T	ITR	SPM	P	T	ITR	SPM
	%	sec	bits/min	sym/min	%	sec	bits/min	sym/min
S01	83	6.20	35.47	6.45	82	5.41	39.89	7.19
S02	100	6.20	50.03	9.68	97	3.44	84.41	16.46
S03	93	6.20	42.67	8.24	91	5.61	45.43	8.71
S04	98	6.20	47.82	9.32	99	3.94	76.86	14.95
S05	94	6.20	43.46	8.42	94	4.14	65.02	12.60
S06	58	6.20	19.87	1.61	76	6.16	30.57	5.05
S07	65	6.20	23.51	2.87	68	6.14	25.41	3.44
S08	92	6.20	41.89	8.06	87	5.26	45.05	8.45
S09	93	6.20	42.67	8.24	85	6.14	37.18	6.88
S10	83	6.20	35.47	6.45	81	5.94	34.96	6.17
S11	90	6.20	40.38	7.71	94	4.64	59.17	11.49
S12	81	6.20	34.15	6.09	81	5.69	36.52	6.45
Avg	86	6.20	38.12	6.93	86	5.21	48.37	8.99

During the free-spelling block, participants were able to express full sentences according to their intentions. Some participants (N = 9) were even able to spell a full sentence without making a single mistake or by performing correctly selected backspaces. Amongst the participants full sentences like “great experiment on Friday thank you” were easily achieved. Results of the free-spelling task are listed in Table 2.

**Table 2:** Free-spelling performance. Performance rates of all individual participants and the grand average during free-spelling. The table lists the total number of K trials (i.e., including backspaces), total number of K’ spelled symbols at the end of the session, number of C correctly spelled symbols, the average time T needed for spelling one symbol (including overhead: inter-trial time), and an estimate of Symbols Per Minute (SPM). Note that only early-stopping was applied, which was trained with a fixed targeted accuracy of 95 percent for all participants. Note that C is an informal measure of correctness.

	Early-Stopping				
	K	K’	C	T	SPM
	#	#	#	sec	sym/min
S01	50	16	16	4.75	4.04
S02	50	50	49	4.36	13.47
S03	50	44	43	4.92	10.48
S04	50	50	50	3.93	15.25
S05	50	38	34	5.12	7.97
S06	50	14	10	6.20	1.94
S07	50	40	0	6.20	0.00
S08	40	26	26	4.56	8.56
S09	29	27	27	4.44	12.59
S10	50	26	13	6.11	2.55
S11	35	27	26	4.08	10.94
S12	50	38	38	5.58	8.17
Avg	46	33	28	5.02	8.00

During debriefing, the participants seemed to be unaware of symbols being encoded by different flash patterns. Furthermore, none of the participants reported the stimulation to be annoying. Instead, participants liked participating in the study and were overall amazed by the ability to spell words solely using brain activity. Most participants tried to influence the performance of the speller by putting more attention to the letter of interest, while ignoring all others. Some participants reported strategies like repeating the symbol of interest in mind.

### Validation of reconvolution

**Fig 9:**
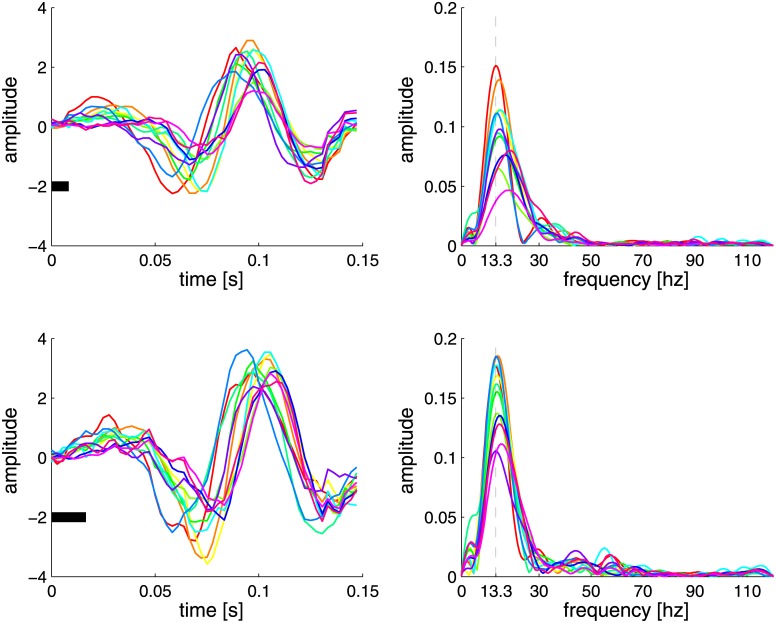
Pulse responses. The spatially filtered pulse responses derived by the estimation step in reconvolution (left) and corresponding zero-padded power spectra (right) are shown for each participant. The top figures show the pulse responses on a short flash, the bottom ones show those for a long flash. The black bars represent the length of a single flash.

Reconvolution can be split into two steps. The first step is estimation, in which pulse responses are derived from the full response. The two pulse responses that are derived from decomposing the signals according to the structure matrix are shown in Fig 9. These resemble a wavelet-like curve: a modulated sine wave with a constant frequency (between 13 and 15 Hertz) and a participant-dependent phase. Also note that the difference between the two pulse responses indicates an enlargement of the amplitude whenever the underlying impulse gets longer. The amplitude and phase of the pulse responses were not significantly correlated with accuracy.

**Fig 10:**
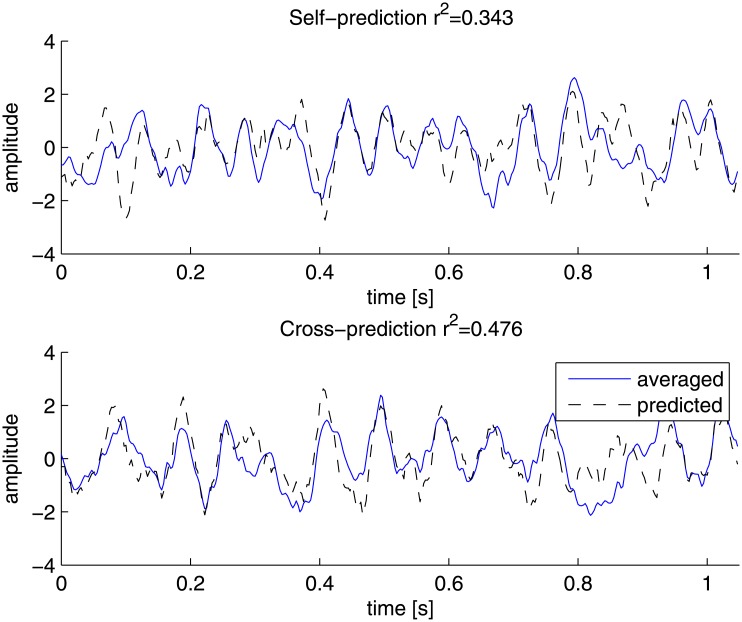
Grand average responses. Grand averages of both spatially filtered ERPs (solid lines) and predicted responses (dashed lines) are shown. The quality of fit by generating the response to the same bit-sequence as reconvolution was trained on is shown at the top (*r* ^2^ = 0.343). The quality of fit by predicting the response to a bit-sequence that was not used during training is shown at the bottom (*r* ^2^ = 0.476).

The second step in reconvolution is generation, in which the estimated pulse responses are superimposed, according to the bit-sequence, and summed to obtain a prediction for the full response. The quality of fit between the original ERP and the predicted ERP for both self-prediction and cross-prediction is shown in Fig 10. The explained variance is computed by performing 10-fold cross-validation. Recall that data was obtained of two different modulated Gold codes during the practice block. By slicing the single-trials to individual code repetitions, 144 trials are obtained. With 10-fold cross-validation responses were predicted with reconvolution using only 14 trials of 1.05 seconds and ERPs were constructed by averaging 130 trials of 1.05 seconds.

**Fig 11:**
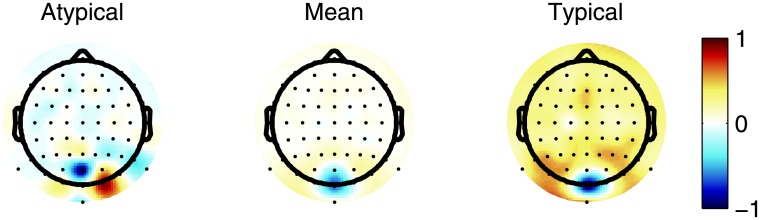
Spatial filters. The grand average spatial filter *W*_*X*_ is depicted (middle). Based on the correlation with this grand average a typical (right, i.e. high correlation) and atypical (left, i.e., low correlation) *W*_*X*_ are shown as well.

The first approach to estimate the predictive power of the reconvolution model is to predict the codes’ own ERP for each channel (i.e., self-prediction). Averaged over participants, the channel-wise explained variance of the first code was on average 4% (min 0%, max 28%) and on average 5% (min 0%, max 30%) for the second code. The channels with highest explained variance were Oz, O1, O2, Iz and POz. By applying CCA to the training fold to derive optimal spatial filters (see Fig 11) and thus to combine channel-wise information, the explained variance is further optimized to an average of 44% (min 37%, max 66%) for the first code and on average 50% (min 37%, max 66%) for the second code. Here, the importance of spatial filtering is clearly visible, because even the best single-channel is outperformed by spatially filtering.

This only proves the ability to predict an already measured ERP. It is more interesting to test generalizability and to estimate the ability to predict unseen classes. We can infer this by cross-predicting the ERP from each code with the reconvolution of the other. Averaged over participants, the channel-wise explained variance of the cross-predicted first code was on average 1% (min 0%, max 10%), and for the second code on average 1% (min 0%, max 11%). By applying CCA, the explained variance is further optimized to an average of 44% (min 30%, max 66%) for the cross-predicted first code and on average 50% (min 37%, max 66%) for the second code.

### Validation of optimizations

By applying reconvolution on the data from the training block, templates were generated for responses to each of the 65 modulated Gold codes in *V*. The cross-correlation matrix of such a subset contained off-diagonal values between -0.38 and 0.42, indicating that there may be room for optimization in picking a subset. An optimal subset of 36 Platinum codes was computed using the clustering technique as outlined above. Among participants different subsets were selected from the set of 65 modulated Gold codes. To illustrate the importance of subset selection, we indicate the ease of confusion (max cross-correlation) of the average of 200 random subset selections and the optimization algorithm. The optimization significantly lowers the maximum cross-correlation between different classes (*p* = 0.001) and the mean correlation (*p* = 0.032), it did not significantly affect the minimum correlation (*p* = 0.051).

Cross-talk may be a problem in BCI performance when responses to non-target neighbouring stimuli leak into the signal. To optimize the layout of the grid we allocated those sequences from the Platinum set that have relatively high cross-correlations to non-neighbouring cells of the 6 by 6 matrix, using the layout optimization algorithm outlined above. The pair-wise cross-correlation of neighbours in the layout was on average -0.04 (min -0.32, max 0.09). As baseline layout selection, we simulated a random process of 200 layouts. With respect to this baseline, the layout optimization significantly lowers the maximum correlation between two neighbours (*p* < 0.001) and the mean correlation (*p* < 0.001). The minimum correlation was not affected (*p* = 0.711).

As shown in Table 1, early-stopping improved the time to make a decision without distortion of the accuracy. A paired t-test was performed to test for a significant effect between the ITR achieved by the fixed-length condition and the early-stopping condition. Early-stopping significantly (*p* = 0.017) improved the communication speed as compared to fixed-length trials in the proposed BBVEP-based speller.

## Discussion

In this study, a 6 × 6 novel BBVEP-based matrix speller is evaluated. The study extends literature by exploring Gold codes for stimulation, instead of m-sequences. Gold codes enable asynchronous BBVEP-based BCI, because Gold codes only show a high auto-correlation at time-lag 0. Specifically, a Gold code is uncorrelated with a any shifted version of itself, as well as with any other code. Thus, in case of Gold codes it is not required to know the exact onset of stimulation. Conversely, in case of one circular-shifted m-sequence a phase-lock is required. Alternatively, a set of different m-sequences does not guarantee optimal cross-correlation. Therefore, this study provides the first steps towards an asynchronous BBVEP-based BCI, without relying on a synchronization process. Additionally, a set of Gold codes contains numerous near-orthogonal codes and therefore allows for direct application to BCIs with a higher number of classes.

A set of Gold codes contains numerous codes, so it becomes unfeasible to generate templates by averaging multiple trials to obtain ERPs. Instead, we proposed a linear generative framework, called reconvolution, with which responses can be predicted. The assumption of linearity is investigated by several other studies (e.g., [17] and [23]). In this study, only two events were used (short and long flashes). In all, reconvolution allows for short training sessions and accurate response-prediction. Specifically, it is shown that reconvolution can explain up to 66% of the variance of spatially filtered ERPs. In this study, reconvolution has been proven to accurately predict responses, because during the testing phase other stimulation sequences were used than during the training phase. These generated responses were well suited to use as templates for a template matching classifier.

A set of Gold codes may contain more codes than is required. We proposed routines to optimize the selection and allocation of a subset of codes to the speller grid, given their predicted responses. Additionally, an early stopping method is proposed that dynamically stops trials when a certain confidence is reached. In this study, the confidence was measured as the difference between the best and second best matching template. Whenever the measured margin was greater than the learned margin, the trial was stopped and the classifier emitted the output. Each of these optimizations were shown to significantly increase discriminability and communication rates.

In summary, on a copy-spelling task, twelve participants reached an average ITR of 48 bits per minute and an SPM of 9 symbols per minute. On a free-spelling task, most participants could spell full sentences, reaching an average of 8 symbols per minute. This communication rate is lower than rates reported in literature (e.g., 144 bits per minute in [11]), but nevertheless an excellent performance when compared to more common stimulation paradigms (e.g., P300-spellers). We propose several ways with which we could increase the performance achieved so far.

First, we clearly separated training from testing by using two sets of modulated Gold codes. The motivation behind this was to ensure reconvolution predicted responses to novel stimuli, which it clearly did well. In principle, there may have been some over-fitting of the training data causing the performance to remain low during testing. However, the validation of reconvolution clearly shows no over-fitting. Specifically, cross-predicting a bit-sequence resulted in similar percentages of explained variance as for self-prediction.

Second, changing stimuli properties could potentially increase performance. In our study we used Gold codes whereas other studies used one m-sequence. M-sequences have near-zero auto-correlation, meaning time-shifted variants are near-orthogonal. One set of Gold codes contains numerous codes that are optimized to a known three-valued cross-correlation, though this cross-correlation is slightly worse than the auto-correlation function of an m-sequence. Thus, the stimulation sequences explored in this study are less orthogonal and could therefore cause a higher level of interference. However, the accuracies achieved in this study imply a clear distinction between codes’ responses up to 100% correct classifications is possible. In addition to the change in stimulus types, we modulated the Gold codes in order to restrict low-frequency content and to limit the events to only short and long flashes. Together with the high stimulation frequency of 120 Hertz this may have affected the brains’ response (e.g., due to a refractory period).

Third, changing the task performed by the participants during the online experiments could potentially increase performance. Participants may have disliked the random copy-spelling task and hence were less motivated to finish the experiment. The more common copy-spelling task in which full natural sentences are spelled, may encourage participants by bringing the task closer to a natural situation; this may increase motivation and attentional resources, contributing to higher accuracies. In addition, an inter-trial interval of 2 seconds was used in our experiment, whereas 0.85 seconds might have been sufficient [11]. Hence, our specific experimental design could be improved for higher accuracies and shorter (inter) trial lengths, which results in a higher ITR.

Fourth, the free-spelling task should best evaluate the practical usage of the system. As is observable from Table 2, for four of twelve participants (participants 1, 6, 7, and 11) only a small part of the selected characters resulted in usable output at the end of the run. For these participants, the majority of selections were either incorrect or used for backspacing. Especially for these participants, but also in general, it remains a trade-off whether or not to utilize a fast but less accurate system, or to improve accuracy by increasing the length of single-trials. It is precisely this objective that an early-stopping algorithm should address dynamically. In this study, the early-stopping algorithm was always calibrated to achieve a 95% targeted accuracy, which was too optimistic for ten out of twelve participants (see Table 1). This may have overcomplicated the estimation of the stopping margins, which as a result becomes an inaccurate measure of confidence.

Future research will aim at improving the methods to meet higher communication rates as reached in literature. Additionally, there is much room for extending BBVEP-based BCI to asynchronous stimulation, other modalities (e.g., [24]), and covert attention (e.g., [25]). Finally, apart from the speller BCI, a few practical applications have been developed and used to demonstrate the system’s feasibility. One of these replicates the fairground can-toss game, in which participants’ gaze is sufficient to make the cans fall down. In the future, we will also test other amplifiers to verify if different applications in the consumer range will become feasible, and to increase the application’s practicality for the end-user (e.g., patients).

## Conclusion

In this study we reported a BBVEP-based BCI using novel stimuli, a generative model and routines to optimize the speller design. More specifically, we presented a paradigm for visual stimulation with so-called modulated Gold codes. The codes were modulated to limit their low frequency content, which also translates them into sequences of only two basic flashes, a short and a long one. We developed a method, called reconvolution, that generates two transient responses, one for each flash. With these transient responses, an independent set of modulated Gold codes (build up from the same two flashes) was predicted. These predictions formed the templates of a template matching classifier, which classifies a new single-trial by maximizing the correlation with the templates. We used Canonical Correlation Analysis to derive optimal spatial filters. Because the set of codes was larger than the needed number of classes, a Platinum subset was selected in which responses to the codes were best distinguished from each other. The codes in this Platinum subset were then optimally allocated to cells in the 6 × 6 speller matrix to minimize cross-talk from neighbouring cells. The data was sliced into small time intervals to derive an early-stopping criterion based on the margin between the best and second best correlation between single-trials and templates.

We tested this BCI setup in an online experiment involving two conditions: fixed-length and early-stopping. In both conditions, a correct classification rate of 86% was achieved. However, in terms of communication rates early-stopping was significantly better (30% on average), achieving an average ITR of 48 bits per minute and SPM of 9 symbols per minute.

The availability of a generative model makes it possible to use a short training session and to optimize the performance of a specific BCI setup without testing all stimulus sequences, which is a great advantage. Reconvolution explained on average 50% up to 66% of the explained variance in spatially filtered responses to both seen and unseen bit-sequences. Together with Gold codes and optimization schemes, a pipeline is provided that can easily be extended to numerous classes and can directly be applied to asynchronous BCI. Both these challenges may be infeasible when m-sequences (i.e., the requirement of a phase-lock) and non-generative methods (i.e., the requirement of long training sessions) are employed.

To conclude, we have shown the importance and performance of a BBVEP-based BCI using Gold codes, a generative model, and several optimization routines, thereby enabling (or restoring) communication and control by brain activity only.
